# The forgotten cohort-lessons learned from prehospital trauma death: a retrospective cohort study

**DOI:** 10.1186/s13049-023-01107-8

**Published:** 2023-08-07

**Authors:** Dominik A. Jakob, Martin Müller, Sebastian Jud, Roland Albrecht, Wolf Hautz, Urs Pietsch

**Affiliations:** 1 Department of Emergency Medicine, Inselspital, Bern University Hospital, University of Bern, 3010 Bern, Switzerland; 2 Department of Anesthesiology and Intensive Care Medicine, Cantonal Hospital St. Gallen, St. Gallen, Switzerland; 3 Swiss Air-Ambulance, Rega (Rettungsflugwacht/Guarde Aérienne), Zurich, Switzerland

**Keywords:** Traumatic cardiac arrest, Prehospital management, Chest decompression, Hemorrhage control, Helicopter emergency medical services

## Abstract

**Background:**

Trauma related deaths remain a relevant public health problem, in particular in the younger male population. A significant number of these deaths occur prehospitally without transfer to a hospital. These patients, sometimes termed “the forgotten cohort”, are usually not included in clinical registries, resulting in a lack of information about prehospitally trauma deaths. The aim of the present study was to compare patients who died prehospital with those who sustained life-threatening injuries in order to analyze and potentially improve prehospital strategies.

**Methods:**

This cohort study included all primary operations carried out by Switzerland's largest helicopter emergency medical service (HEMS) between January 1, 2011, and December 31, 2021. We included all adult trauma patients with life-threatening or fatal conditions. The outcome of this study is the vital status of the patient at the end of mission, i.e. fatal or life-threatening. Injury, rescue characteristics, and interventions of the forgotten trauma cohort, defined as patients with a fatal injury (NACA score of VII), were compared with life-threatening injuries (NACA score V and VI).

**Results:**

Of 110,331 HEMS missions, 5534 primary operations were finally analyzed, including 5191 (93.8%) life-threatening and 343 (6.2%) fatal injuries. More than two-thirds of patients (n = 3772, 68.2%) had a traumatic brain injury without a significant difference between the two groups (*p* > 0.05). Thoracic trauma (44.6% vs. 28.7%, *p* < 0.001) and abdominal trauma (22.2% vs. 16.1%, *p* = 0.004) were more frequent in fatal missions whereas pelvic trauma was similar between the two groups (13.4% vs. 12.9%, *p* = 0.788). Pneumothorax decompression rate (17.2% vs. 3.7%, *p* < 0.001) was higher in the forgotten cohort group and measures for bleeding control (15.2% vs. 42.7%, *p* < 0.001) and pelvic belt application (2.9% vs. 13.1% *p* < 0.001) were more common in the life-threating injury group.

**Conclusion:**

Chest decompression rates and measures for early hemorrhage control are areas for potential improvement in prehospital care.

**Supplementary Information:**

The online version contains supplementary material available at 10.1186/s13049-023-01107-8.

## Background

Although rates are declining for nearly all injuries worldwide, trauma remains an important cause of mortality [1]. Each year there are still five to six million deaths as a result of trauma. In particular, traumatic injury remains one of the leading causes of death in the population aged five to twenty-nine years, with males significantly more affected than females [2–5].

The primary cause of early death in trauma is dominated by central nervous system injury and exsanguination [6]. The latter, in particular, is potentially preventable with optimized trauma care. This is also evident from autopsy studies of trauma-related deaths, which indicated that 15–19% of deaths are potentially preventable [7–9]. These studies also identified suboptimal care in 65% of fatal trauma, and medical interventions that were delayed in 58% of the cases.

Prehospital treatment in trauma is a crucial part of the rescue chain and an important factor in determining patient outcomes. Helicopter emergency medical services (HEMS) is a substantial part of prehospital trauma care in most Western countries [10–12]. Particularly in alpine areas, HEMS has been shown to shorten rescue times and was associated with a lower mortality in trauma [13–19]. National trauma registries have been established worldwide to analyze epidemiology, injury patterns, treatment, and outcomes of severely injured trauma patients. However, most national trauma registries, such as the National Trauma Database in the United States [20], and also the Swiss trauma registry [21], do not include patients who died prehospitally and that were not being transferred to a hospital. As a result, there is a complete lack of information about patients who died prehospital. This information would be important to gain in-depth knowledge about this important group, particularly to improve prehospital trauma care [22–27]. The aim of the present study was to compare patients who died before hospital admission with those who sustained life-threatening injuries in HEMS missions.

## Methods

### Study design and Setting

This retrospective observational cohort study included all primary rescue missions of at least life-threatening injured trauma patients of the Swiss Air-Ambulance Rega, between January 1, 2011 and December 31, 2021 (11 years). Rega is providing 24/7 physician-staffed HEMS for prehospital retrievals (primary missions) and interfacility transfers (secondary missions) in Switzerland and carries out approximately 16,000 HEMS missions yearly, two-third of these are primary missions (Additional file 1: Table S1A) [10, 28]. This study is reported in accordance with the STROBE statement [29].

### Eligibility criteria

All missions in the study period conducted by Rega were eligible for analysis. Excluded were missions that were (i) secondary, (ii) not related to trauma, or had a (iii) NACA score of <5. We also excluded operations with missing on-scene time or those exceeding 240 min (excluding incomplete/poorly documented missions), as well as those that did not involve any advanced medical interventions (Additional file 1: Table S1B) such as missions solely focused on recovering bodies or determining death.

### Descriptive parameters and potential predictors of survival

The following potential predictors of survival were extracted from the electronic medical record system: (i) *mission & rescue characteristics, *(ii) *demographics, *(iii)* type of injury, *(iv) *injury characteristics* and (v) mission d*urations* (Additional file 1: Table S1C). The following additional baseline characteristics were obtained: breathing or heart actions, circulation,and Glasgow Coma Scale (GCS) [30].

Furthermore, specific medical interventions on scene were extracted, i.e. actions regarding (i) *airway, *(ii) *(hemodynamic) monitoring, *(iii) *resuscitation, *(iv) *pneumothorax decompression, *(v)* bleeding control and *(vi) *drug application* (Additional file 1: Table S1D).

### Stratification of the study population

The study population was stratified by the vital status of the patient at the end of mission, i.e. dead or alive. The term "fatal mission” is defined as missions in which patients were pronounced dead at the scene and assigned a NACA score of VII [31]. Such cases are included as the “forgotten trauma cohort”, which comprises patients who have not survived (short-term) their injuries despite the deployment of rescue services. The term “life-threatening mission/ non-fatal mission” was defined as a NACA score of V or VI [31].

### Data sources

Mission details are systematically and prospectively recorded in the Rega database by different members of the HEMS crew, including physicians, paramedics and pilots. The information captured includes a wide range of mission and rescue characteristics, such as time, geocoordinates, and aviation details. In addition, the database includes extensive patient-related information, such as demographics, type of injury, baseline monitoring, and any interventions performed during the mission. The variables studied were extracted out of the database.

### Statistical methods

The statistical analysis was performed with Stata® 16.1 (StataCorp, The College Station, TX, USA). For descriptive analysis, continuous variables were presented as median with interquartile range (IQR) as most of the continuous variables were not normally distributed. Categorical variables were reported as counts and percentages (%) for each level of the variable. The medical interventions performed in the two study groups (fatal vs. non-fatal missions) were shown in a waffle chart.

We investigated predictors potentially associated with fatal outcomes in HEMS missions through univariable and multivariable logistic regression analyses. Odds ratios (OR) with 95% confidence intervals (CI) were shown as effect sizes. Factors that showed at least very weak evidence for an association (*p* < 0.2) were included in multivariable logistic regression analysis. Non-significant predictors (*p* < 0.05) were excluded stepwise to obtain a parsimonious final model. Accuracy of the resulting model was assessed with the AUROC (values > 0.7 are considered as an acceptable accuracy). The effect sizes of all variables included in the final multivariable model were visualized with a regression coefficient plot, OR and 95% CI. Multicollinearity analysis of the final model was conducted using the—*Collin*—command to examine the correlation between variables. A variance inflation factor of less than 2.5 was used as the threshold for identifying problematic levels of multicollinearity [32].

For sensitivity analysis, prehospital time variables were excluded as predictors from the final model as some might argue that they are not predictors but rather a consequence of fatal injuries.

In a supplemental analysis, baseline characteristics of both non-fatal and fatal groups were compared without excluding patients who did not receive any advanced medical action.

## Results

### Study population

**Fig. 1:**
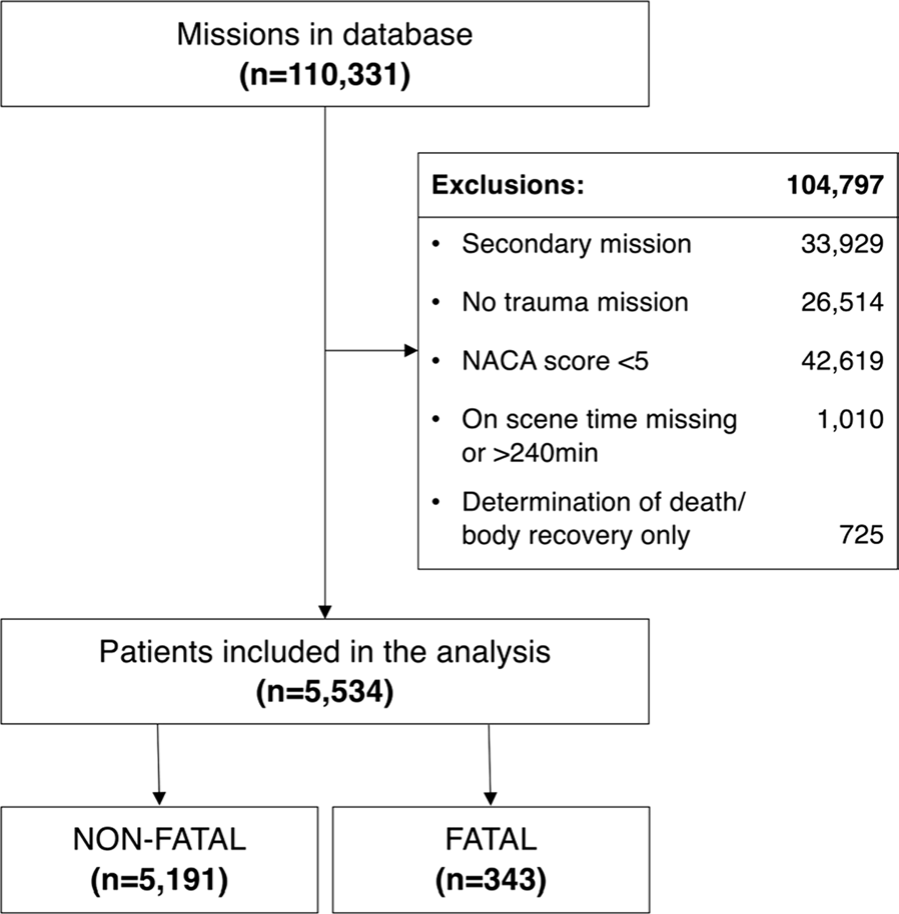
Study flow chart. Abbreviation: NACA National Advisory Committee for Aeronautics score

In total, 110,331 missions in the database over the 11 years study period were screened for eligibility. Excluded were 33,929 secondary missions, 26,514 non-trauma missions, 42,619 trauma missions with a NACA score lesser than five, 1010 missions with missing data or more than 4 h on scene time, and 725 missions without any advanced medical action being performed. Of the 5534 missions included, 5191 (93.8%) were documented as non-fatal missions (NACA V/VI) and 343 (6.2%) as fatal missions (NACA VII), see Fig. 1.

### Baseline characteristics and univariable analysis

The baseline characteristics of the total study population as well as in the study groups are shown in Table 1. Late-night missions and female gender were more often found in life-threatening (22.0% & 26.6%) than fatal missions (13.1% & 19.2%, *p* < 0.001, *p* = 0.002). The age distribution was similar between the two groups (*p* = 0.445) with a median of 49 (IQR 27–65) years in the total group. A winch rescue, indicating difficult terrain, was performed more often in fatal missions (12% vs. 6.0%, *p* < 0.001). Initial vital signs (breathing, pulse, vigilance) were considerably more often found in non-fatal missions. The most common types of injury in life-threatening missions were household (27.6%), MVC (14.7%), and work-related (12.3%). In fatal missions MCC (17.2%), work-related (15.2%), and MVC (13.4%) were the most common. Winter sports accidents were more among fatal injuries(10.2% vs. 3.9%, *p* < 0.001).

**Table 1:** Baseline characteristics of the 5534 included fatal (n = 343) and non-fatal (n = 5191) missions as well as univariable logistic regression to predict fatal injuries

	Total (n = 5534)		Life-threatening injury (n = 5191)		Fatal injury (n = 343)		Odds ratio	(95% CI)	*p*-value
Mission and rescue details									
*Season of mission, n (%)*									
Winter	1005	[18.2]	942	[18.1]	63	[18.4]	1.00	(baseline)	
Spring	1471	[26.6]	1368	[26.4]	103	[30.0]	1.13	(0.81; 1.56)	0.474
Summer	1928	[34.8]	1822	[35.1]	106	[30.9]	0.87	(0.63; 1.20)	0.396
Fall	1130	[20.4]	1059	[20.4]	71	[20.7]	1.00	(0.71; 1.42)	0.989
WE mission, n (%)	1783	[32.2]	1659	[32.0]	124	[36.2]	1.21	(0.96; 1.51)	0.108
Late/night mission (20:00–07:59), n (%)	1188	[21.5]	1143	[22.0]	45	[13.1]	0.53	(0.39; 0.74)	< 0.001
Winch rescue, n (%)	353	[6.4]	312	[6.0]	41	[12.0]	2.12	(1.50; 3.00)	< 0.001
*Demographics*									
Age [years]^+^, med (IQR)	49	[27; 65]	49	[27; 66]	48	[28; 62]	1.00	(0.99; 1.00)	0.445
Age > 65y, n (%)	1382	[25.0]	1309	[25.2]	73	[21.3]	0.80	(0.61; 1.05)	0.104
Female gender, n (%)	1447	[26.1]	1381	[26.6]	66	[19.2]	0.66	(0.50; 0.87)	0.003
*Type of injury*									
MVC	810	[14.6]	764	[14.7]	46	[13.4]	0.90	(0.65; 1.24)	0.507
MCC	694	[12.5]	635	[12.2]	59	[17.2]	1.49	(1.11; 2.00)	0.007
Bike	444	[8.0]	425	[8.2]	19	[5.5]	0.66	(0.41; 1.06)	0.083
CVP	314	[5.7]	298	[5.7]	16	[4.7]	0.80	(0.48; 1.34)	0.405
Sky activity	106	[1.9]	94	[1.8]	12	[3.5]	1.97	(1.07; 3.62)	0.030
Hiking/climbing	190	[3.4]	168	[3.2]	22	[6.4]	2.05	(1.30; 3.24)	0.002
Winter sports	237	[4.3]	202	[3.9]	35	[10.2]	2.81	(1.93; 4.09)	< 0.001
Work-related	690	[12.5]	638	[12.3]	52	[15.2]	1.28	(0.94; 1.73)	0.120
Household	1470	[26.6]	1432	[27.6]	38	[11.1]	0.33	(0.23; 0.46)	< 0.001
*Injury characteristics*									
TBI, n (%)	3772	[68.2]	3553	[68.4]	219	[63.8]	0.81	(0.65; 1.02)	0.077
Thoracic trauma, n (%)	1642	[29.7]	1489	[28.7]	153	[44.6]	2.00	(1.60; 2.50)	< 0.001
Abdominal trauma, n (%)	912	[16.5]	836	[16.1]	76	[22.2]	1.48	(1.14; 1.93)	0.004
Pelvic trauma, n (%)	716	[12.9]	670	[12.9]	46	[13.4]	1.05	(0.76; 1.44)	0.788
Upper extremity trauma, n (%)	897	[16.2]	859	[16.5]	38	[11.1]	0.63	(0.44; 0.89)	0.008
Lower extremity trauma, n (%)	1130	[20.4]	1063	[20.5]	67	[19.5]	0.94	(0.72; 1.24)	0.674
*Durations*									
Response time* [min], med (IQR)	19	[15; 24]	19	[15; 24]	17	[13; 22]	0.90	(0.81; 1.01)	0.076
On scene time* [min], med (IQR)	28	[22; 39]	28	[21; 37]	51	[35; 69]	1.62	(1.54; 1.70)	< 0.001

*CVP* car versus pedestrian, *GCS* Glasgow Coma Scale, *IQR* interquartile range, *MCC* motorcycle crash, *med* median, *min* minutes, *MVC* motor vehicle crash, *TBI* traumatic brain injury, *WE* weekend

^+^For the odds ratio: per 1 year increase

*For the odds ratio: per 10 min increase

More than two-thirds of the missions (n = 3772, 68.2%) had a suspected TBI, and nearly one-third (n = 1642, 29.7%) of all missions had a documented thoracic trauma. Thoracic trauma (44.6% vs. 28.7%, *p* < 0.001) and abdominal trauma (22.2% vs. 16.1%, *p* = 0.004) were more frequent in fatal missions compared to life-threatening missions whereas pelvic trauma was similar between the two groups (13.4% vs. 12.9%, *p* = 0.788).

The median response time was 2 min shorter in fatal injuries [19 (IQR 15–24) minutes vs. 17 (IQR 13–22) minutes, *p* = 0.076] and on-scene time was twice as long [51 (IQR 35–69) minutes vs. 28 min, (IQR 22–39) minutes, *p* < 0.001]. Additional baseline characteristics are shown in Additional file 1: Table S2. Descriptive baseline characteristics were similar if all fatal missions were included (see Additional file 1: Table S3).

### Performed medical interventions

**Fig. 2:**
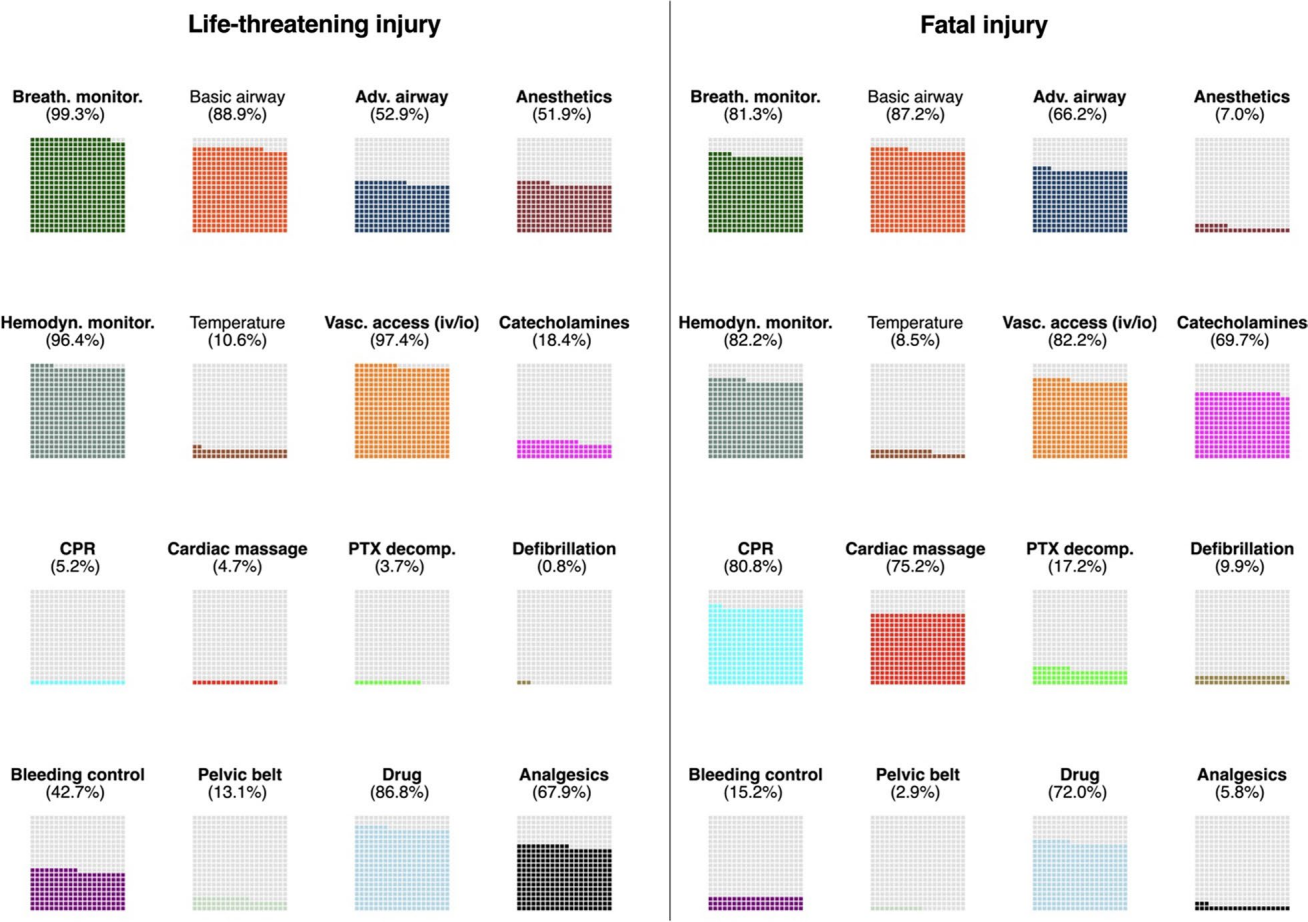
Waffle chart of medical interventions in 5191 life-threatening (left) and 343 fatal (right) injuries. Medical interventions in bold are significant (< 0.001) between the groups. Abbreviations: adv., advanced; breath., breathing; CPR, cardiopulmonary resuscitation; decomp., decompression; monitor., monitoring, PTX, pneumothorax, vasc., vascular

Medical interventions performed prehospitally are shown in Fig. 2 (and Additional file 1: Table S4). Except for basic airway management and temperature control, all medical interventions differed significantly between the two groups (*p* < 0.001). The largest differences were identified in rates of catecholamine administration (69.7% vs. 18.4%), CPR (80.8%vs. 5.2%), defibrillation (9.9% vs. 0.8%), and advanced airway management (66.2% vs. 52.9%), all with higher rates in the “forgotten trauma cohort” group.

In total, 17.2% of the forgotten cohort received some form of pneumothorax decompression compared to 3.7% in the life-threatening group. In the forgotten cohort, measures for bleeding control were performed in 15.2% and a pelvic belt was used in 2.9%. Both interventions were more frequent in patients with life-threatening injuries (bleeding control in 42.7% and use of pelvic belt in 13.1%).

### Multivariable analysis

**Fig. 3:**
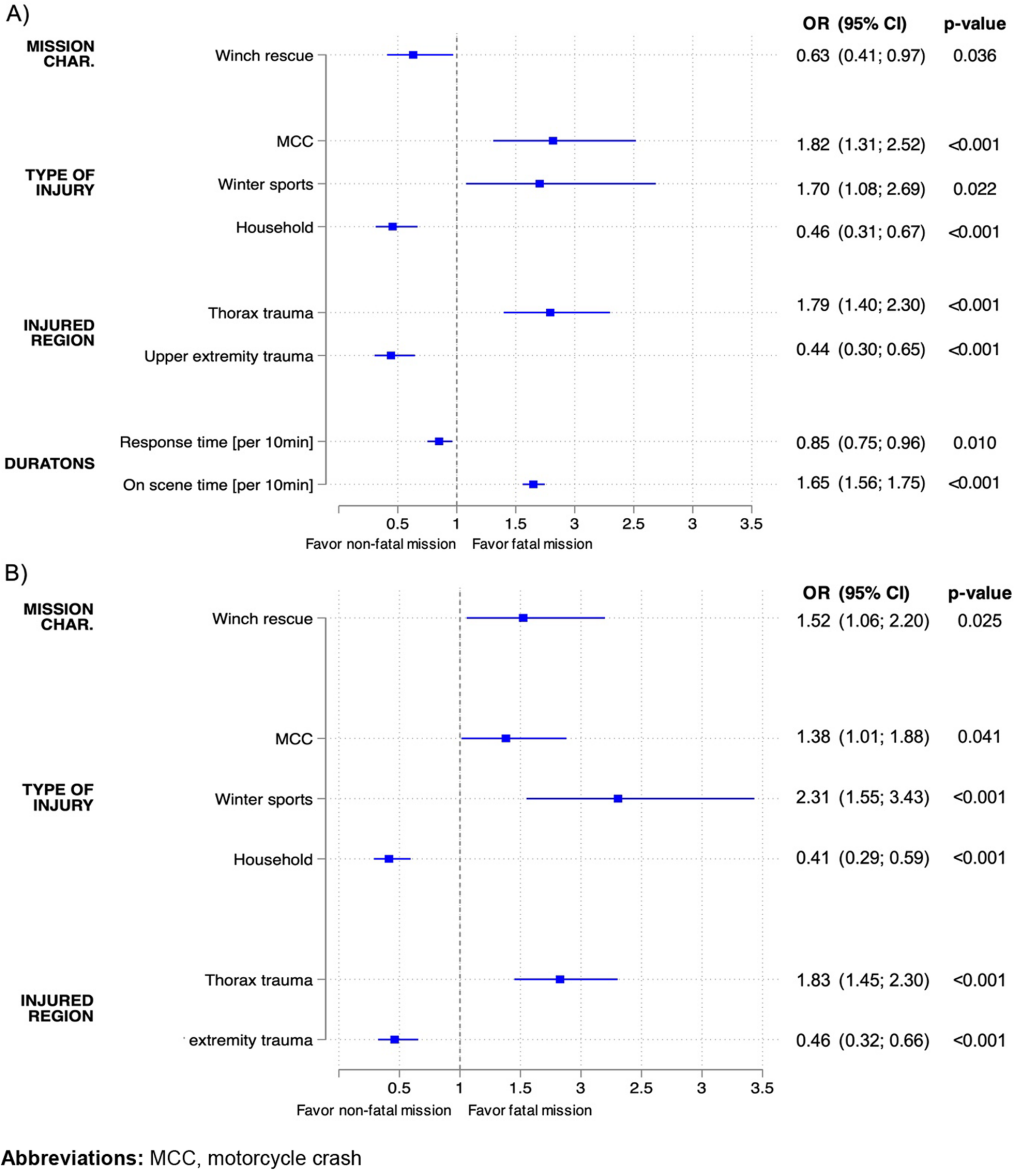
Multivariable analysis of significant predictors for a fatal mission (total missions: n = 5534), **A** final model, **B** sensitivity analysis, excluding prehospital durations from the final model. Abbreviations: MCC, motorcycle crash

The final model to predict fatal outcome was obtained by excluding stepwise all non-significant factors (*p* ≥ 0.05) from the identified potential predictors through univariable analysis (Fig. 3).

The final multivariable analysis had a good performance with an AUROC of 0.809. Risk factors for fatal injuries were MCC and winter sport injuries and thoracic trauma. Increasing time on scene was also associated with a fatal outcome. Household injuries, upper extremity trauma, and increasing response time were predictive of non-fatal missions (see Fig. 3a). Excluding prehospital durations from the model lead to a change of the direction of the effect of winch rescue, identifying it as a risk factor, while other effect sizes were similar in magnitude and direction (Fig. 3b).

## Discussions

Little is known about trauma patients who die before hospital admission, because these patients are typically not included in clinical registries. As a result, there is a lack of knowledge about the circumstances leading to prehospital trauma death and the interventions performed professionally to save these patients [22, 27, 33].

The present study compared patient characteristics and interventions for fatal injuries with those suffering life-threatening injuries to identify potential areas for improvement in prehospital strategies. Overall, 5,534 primary missions including 93.8% life-threatening and 6.2% fatal missions of the largest Swiss HEMS over a 11 years study period were analyzed. MCC, thoracic trauma and increasing on scene time were identified as independent risk factors for fatal injuries. These findings are already well known in literature to be associated with severe injuries and increased mortality [34–37]. The identified factors associated with a decreased mortality were somehow counterintuitive. Upper extremity injuries might reflect the patients’ ability to successfully protect against more severe injuries in the event of an accident and were therefore identified as protective for mortality. Household injures as protective factor might be the expression that predictive factors were identified in the most severely injured and fatal cohort. Winch rescue is considered as part of the time on scene, therefore excluding time on scene from the regression model leads to a change in the direction of the winch rescue effect. The mortality benefit associated with increased response time cannot be readily explained but might be an expression of a selection bias. In line, medical interventions such as measures for CPR, catecholamine administration and prolonged prehospital times could also be considered more as consequence than a predictor of fatal outcome.

It is well known that hemorrhage and central nervous system injury predominate among the causes of prehospital death [6, 38, 39]. Bleeding control in particular is essential to improve outcomes in patients with traumatic injuries as reported in different studies and guidelines [40–43]: for example, in a recent study of stab wounds in urban areas, Vulliamy et al. recommended expanding initiatives that promote bystander-delivered hemorrhage control of extremity injuries to improve outcomes for these patients [44–46]. Studies in combat injuries also showed that up to 15% of the deaths were potentially survivable and especially highlighted the need for improvements in truncal hemorrhage control [39, 47, 48]. Acute blood loss has also been shown to be the major cause of death within 24 h in blunt trauma [49–54], indicating the potential for improved management strategies.

One promising approach for the early management of bleeding could be the transfusion of whole blood and blood products in the prehospital setting [55–57]. In particular, combat studies have highlighted improved survival rates for patients who received prehospital blood products [58, 59]. In Switzerland, blood products are not regularly used in the prehospital setting and whole blood is not available. A recently published study evaluated the time to resuscitation in predominantly blunt trauma patients with hemorrhagic shock [50]. An early resuscitative intervention in this study was defined as plasma, packed red blood cell or tranexamic acid administration in the field or within 90 min of trauma center arrival. The results showed that every 1-min increase in time to early resuscitative intervention was associated with 2% increase in the odds of 30-day mortality and 1.5% increase in odds of 24-h mortality.

Our findings also indicate that patients who died prehospitally had more frequent chest and abdominal injuries compared to patients who survived to hospital admission. Both of these injuries are frequently associated with fatal hemorrhage. On the other hand, our results also show that the interventions performed to control hemorrhage were almost three times as common for life-threatening injuries (42.7%) compared to fatal injuries (15.2%). In this regard, it was also noticeable that a pelvic trauma was suspected in 13.4% of all patients who died prehospital, but a pelvic belt was applied in less than 3%. These findings may indicate an area for improvement in severely injured patients. However, it must also be noted that in particular, prehospital torso hemorrhage control in trauma is a huge challenge.

A possible approach to address these types of bleedings could be the use of prehospital REBOA (resuscitative endovascular balloon occlusion of the aorta) [60, 61]. A recently published systematic review evaluated the role of prehospital REBOA and concluded that the procedure was feasible in 68%-100% of trauma patients. However, survival and complication rates in REBOA varied widely [62, 63]. Furthermore, the authors also emphasizes that the procedure requires a coordinated and integrated emergency health care system with a well-trained and equipped team. All these challenges must be overcome, and prospective data demonstrating the true benefits of prehospital REBOA are needed before the procedure can be widely implemented. Particularly in the prehospital setting, the potential benefits of a REBOA must be balanced against the extended on-scene time.

Another area for potential improved management is the prehospital chest decompression rate. Pneumothoraxes, in particular tension pneumothoraces, are well recognized causes of preventable deaths in trauma patients [7, 9, 47]. A population-based study by Bartolome et al. estimated a prevalence of pneumothorax in one of five major trauma victims found alive [64]. Literature regarding the overall incidence of tension pneumothorax varies widely and dependent highly on the trauma mechanism [65–67]***.*** For example, a combat study conducted during the Vietnam War found that tension pneumothorax was the attributed cause of death in approximately 3–4% of the cases [67]. In the present study, thoracic injuries were reported in almost 45% of patients who died prehospital*.* An indeterminate proportion of these individuals might have experienced tension pneumothorax, which could have potentially been relieved through chest decompression**.** In addition, thoracic trauma was identified as an independent risk factor for fatal injury. Despite these facts, less than 20% of the patients who died prehospital underwent any chest decompression, even though 80% of the “fatal cohort” underwent CPR. Chest decompression in traumatic (peri-)arrest situation is essential as an untreated tension pneumothorax will inevitably lead to death caused by impaired cardiac filling, reduced venous return due to mediastinal shift, and elevated pulmonary vascular resistance caused by hypoxemia [68–70]. As a consequence, chest decompression in traumatic cardiac (peri-)arrest is stated in all recent CPR guidelines, which was also highlighted in a recent study characterizing fatal blunt injuries [33, 71, 72]. In the present study the reasons for the low pneumothorax decompression rate can only be a matter of speculation. A recently published qualitative study evaluating decision-making in prehospital TCA revealed that not all prehospital providers feel sufficiently trained to perform prehospital interventions on patients with TCA [73]. Although it must be assumed that the HEMS physicians, which are all board certificated in anesthesiology and prehospital emergency medicine, should be adequately trained for these procedures. In summary, however, more responsive pneumothorax decompression measures may improve outcomes. A promising option in the future is the Point-of-care ultrasound as a valuable tool for narrowing the differential diagnosis for reversible causes of TCA so that appropriate therapies like chest decompression can be initiated. Therefore, focused prehospital ultrasound has the potential to further refine our differential diagnosis and tailor therapies for successful resuscitation [33, 74, 75].

In addition, it was interesting that patients in the “forgotten cohort” group received more likely an advanced airway management (i.e., intubation, surgical airway, mechanical ventilation) compared to patients who were severely injured. It is well known that intubation might be a life-saving procedure for patients who fail to maintain a patent airway or are unable to oxygenate and ventilate adequately. However, the potential benefit is also associated with risks. Difficult or failed endotracheal intubation may cause hypoxemia, aspiration, and hypotension [76]. Especially in patients with hemorrhagic shock, intubation often leads to cardiac arrest. The underlying mechanisms are loss of sympathetic tone after induction medication, positive pressure ventilation with reduced cardiac output, and an expanding hematoma resulting from the loss of muscle tone due to paralyzing drugs. Therefore, a recently published study suggested that for patients with hemorrhagic shock who do not have a compromised airway and who are able to maintain adequate oxygen saturation, a strategy of delayed intubation should be strongly encouraged [77].

The present study allowed to evaluate prehospital traumatic deaths in HEMS. These patients are barely addressed in the scientific medical literature, although HEMS are often dispatched to patients with major trauma, because they can provide treatments and advanced interventions in the prehospital environment that have the potential to increase survival [78, 79]. At HEMS in Switzerland, a physician is always part of the team. However, it is important to note that in the event of serious accidents in Switzerland, ground-based emergency medical service (GEMS) also involve a physician with equipment similar to that provided by HEMS [80]. HEMS missions for severely injured patients are therefore comparable to GEMS, both in terms of personnel and medical equipment. Consequently, findings from HEMS missions can be generalized for prehospital care of severely injured patients, at least in Switzerland. A major advantage of the present study is the large number of consecutively included trauma patients who died prehospital or had life-threatening injuries. A particular strength was our study design including patients in the fatal group only when advanced medical interventions were documented. Missions solely focused on recovering bodies or determining death were excluded. This guaranteed that only patients who could potentially survive were considered for analysis in the fatal group. However, several limitations need to be addressed: First, the cause of death was deduced from the suspected injuries and was not evaluated by autopsy. Second, circumstances why a prehospital treatment was or was not initiated are not described in the database. Third, the data contained in the database were mostly classified according to the judgment of the physician present on scene. For example, objective parameters for injury classification, such as radiological imaging, could not be used. Furthermore, the decision to initiate advanced medical measures was ultimately made by the physician present. It is possible that some of the patients who received advanced medical actions were already dead for a prolonged period without any chance of survival. A possible selection bias might therefore be present. Also, the NACA score is only recorded once per mission and judges only the most critical period during the mission. Thus, the NACA score does not reflect any improvement in the patient due to therapeutic interventions performed like early on-scene treatment (e.g. airway obstruction, tension pneumothorax, ana-phylactic shock). Lastly, all missions were completed by REGA using the same treatment standards and many missions in this analysis involved patients undertaking recreational activities in the mountains in summer and winter (e.g. skiing, hiking or climbing). Our findings are therefore not necessarily transferable to other countries [81, 82].

## Conclusions

The results provided have implications for prehospital strategy, quality improvement and public health prevention measures. In particular, chest decompression rates and measures for early hemorrhage control are areas for potential improvement in prehospital trauma care.

### Supplementary Information

**Additional file 1**. Table S1–S4.

## Data Availability

The datasets generated and/or analysed during the current study are not publicly available, but are available from the corresponding author on reasonable request.

## Abbreviations

AUROC: Area under the curve
GCS: Glasgow Coma Scale
CPR: Cardiopulmonary reanimation
CVP: Car versus pedestrian
GEMS: Ground-based emergency medical service
HEMS: Helicopter emergency medical service
IQR: Interquartile range
MCC: Motor cycle crash
MVC: Motor vehicle crash
NACA: National Advisory Committee for Aeronautics
PTX: Pneumothorax
REBOA: Resuscitative endovascular balloon occlusion of the aorta
TBI: Traumatic brain injury
TCA: Taumatic cardiac arrest
WE: Weekend
