# Supplementary material for: The forgotten cohort-lessons learned from prehospital trauma death: a retrospective cohort study
Source: Scand J Trauma Resusc Emerg Med. 2023 Aug 7;31:37. doi: 10.1186/s13049-023-01107-8 (PMC10405424; doi:10.1186/s13049-023-01107-8)
Supplement: Supplementary file 1 — Additional file 1. Table S1–S4. [file 13049_2023_1107_MOESM1_ESM.docx]

**Supplement tables**

Supplement 1. Detailed definition of A) Details for the Swiss Air-Ambulance Rega, B) Advanced medical actions, C) Potential predictors, and D) Specific medical interventions on scene

A) Details for Swiss Air-Ambulance Rega

Largest organization in Switzerland, operating 14 helicopter bases throughout the country

Any location in its operational area is reachable within 15 minutes of flight on a 24/7 availability, as far as flight is possible.

All helicopters are equipped with a certified rescue hoist and avionics that allow visual flight, instrument flight, as well as flight with and without night vision goggles

The crew includes a HEMS physician, a pilot, and a paramedic, who also serves as a technical crew member and hoist operator

In some missions in rural mountain terrain, a rescue specialist is added to the crew. In these situations, the HEMS physician is winched down to the emergency site either first or after assessment by the rescue specialist

B) Advanced medical actions were defined as any of the following:

airway/breathing action: intubation, mask/mechanical ventilation, surgical airway, monitoring of end-tidal CO2, insertion chest drainage, needle or finger thoracostomy

CPR action: cardiac massage, defibrillation, CPR devices active/activated

establishment of vascular access: peripheral, central, intraosseous

special drug application: catecholamines, muscle relaxants or analgesics

other advanced action: bleeding control (see below) or reduction of luxated joints

C) Details on potential predictors of survival:

Mission & rescue characteristics: season, day of week, weekend (we) and night mission (20:00-07:59) and if a winch was used for rescue

Demographics: age in years, old age (>65 years) and gender

Type of injury in the categories: car versus pedestrian (CVP) injury, motorcycle crash (MCC), motor vehicle crash (MVC), bike crash, sky activity injury, hiking/climbing injury, winter sports, household, work-related, and other

Injury characteristics: thorax, abdomen, pelvis, lower/upper extremity trauma and traumatic brain injury (TBI)

Durations: response time in minutes (defined as the time from call for assistance until arrival on scene) and time on scene in minutes (from arrival to leaving of the scene)

D) Specific medical interventions on scene were extracted:

airway: basic (i.e., oxygenation or bag mask ventilation) or advanced (i.e., intubation, surgical airway, mechanical ventilation)

(hemodynamic) monitoring: ECG, (non-) invasive blood pressure, central venous pressure, and temperature

resuscitation: cardiac massage and defibrillation

pneumothorax decompression: needle decompression, thorax drainage or thoraco(s)tomy

bleeding control: hemostasis, dressing, reduction, wound care, tranexamic acid, or pelvic belt administration

drugs: analgesics, catecholamines, or anesthetic drug (muscle relaxants, narcotics, or sedatives)

Supplement 2. Additional baseline characteristics

|  | Total (n=5534) | | Life-threatening injury (n=5191) | | Fatal injury (n=343) | |
| --- | --- | --- | --- | --- | --- | --- |
| MISSION & RESCUE DETAILS | |  |  |  |  |  |
| Day of week of mission, n (%) | |  |  |  |  |  |
| Sunday | 832 | [15.0] | 773 | [14.9] | 59 | [17.2] |
| Monday | 711 | [12.8] | 677 | [13.0] | 34 | [9.9] |
| Tuesday | 712 | [12.9] | 669 | [12.9] | 43 | [12.5] |
| Wednesday | 740 | [13.4] | 697 | [13.4] | 43 | [12.5] |
| Thursday | 775 | [14.0] | 726 | [14.0] | 49 | [14.3] |
| Friday | 813 | [14.7] | 763 | [14.7] | 50 | [14.6] |
| Saturday | 951 | [17.2] | 886 | [17.1] | 65 | [19.0] |
| TYPE OF INJURY | |  |  |  |  |  |
| Injury type, n (%) | |  |  |  |  |  |
| MVC | 810 | [14.6] | 764 | [14.7] | 46 | [13.4] |
| MCC | 694 | [12.5] | 635 | [12.2] | 59 | [17.2] |
| Bike | 444 | [8.0] | 425 | [8.2] | 19 | [5.5] |
| AVP | 314 | [5.7] | 298 | [5.7] | 16 | [4.7] |
| Sky activity | 106 | [1.9] | 94 | [1.8] | 12 | [3.5] |
| Hiking/Climbing | 190 | [3.4] | 168 | [3.2] | 22 | [6.4] |
| Winter sports | 237 | [4.3] | 202 | [3.9] | 35 | [10.2] |
| Work-related | 690 | [12.5] | 638 | [12.3] | 52 | [15.2] |
| Household | 1470 | [26.6] | 1432 | [27.6] | 38 | [11.1] |
| Other | 579 | [10.5] | 535 | [10.3] | 44 | [12.8] |
| PATIENT CHARACT. | |  |  |  |  |  |
| Breathing, n (%) | 3443 | [62.2] | 3436 | [66.2] | 7 | [2.0] |
| Heart actions, n (%) | 4783 | [86.4] | 4770 | [91.9] | 13 | [3.8] |
| Circulation, n (%) | 4270 | [77.2] | 4260 | [82.1] | 10 | [2.9] |
| GCS, med (IQR) | 7 | [3.0; 14.0] | 8 | [3.0; 14.0] | 3 | [3.0; 3.0] |
| Docum. GCS<9, n (%) | 2935 | [53.0] | 2603 | [50.1] | 332 | [96.8] |

Abbreviations: AVP, auto versus pedestrian; GCS, Glasgow Coma Scale; MCC, motorcycle crash; MVC, motor vehicle crash

Supplement 3. Baseline characteristics (including all death)

|  | Total  (n=6259) | | Life-threatening injury  (n=5250) | | Fatal injury  (n=1009) | | P-value |
| --- | --- | --- | --- | --- | --- | --- | --- |
| MISSION & RESCUE CHARACTERISTICS |  |  |  |  |  |  |  |
| Season of mission, n (%) |  |  |  |  |  |  |  |
| Winter | 1094 | [17.5] | 957 | [18.2] | 137 | [13.6] |  |
| Spring | 1665 | [26.6] | 1382 | [26.3] | 283 | [28.0] |  |
| Summer | 2235 | [35.7] | 1841 | [35.1] | 394 | [39.0] |  |
| Fall | 1265 | [20.2] | 1070 | [20.4] | 195 | [19.3] | 0.001 |
| Day of week of operation, n (%) |  |  |  |  |  |  |  |
| Sunday | 949 | [15.2] | 784 | [14.9] | 165 | [16.4] |  |
| Monday | 811 | [13.0] | 687 | [13.1] | 124 | [12.3] |  |
| Tuesday | 807 | [12.9] | 679 | [12.9] | 128 | [12.7] |  |
| Wednesday | 835 | [13.3] | 706 | [13.4] | 129 | [12.8] |  |
| Thursday | 875 | [14.0] | 734 | [14.0] | 141 | [14.0] |  |
| Friday | 884 | [14.1] | 765 | [14.6] | 119 | [11.8] |  |
| Saturday | 1098 | [17.5] | 895 | [17.0] | 203 | [20.1] | 0.087 |
| WE mission, n (%) | 2047 | [32.7] | 1679 | [32.0] | 368 | [36.5] | 0.005 |
| Late/night mission (20:00-07:59), n (%) | 1263 | [20.2] | 1150 | [21.9] | 113 | [11.2] | <0.001 |
| Winch rescue, n (%) | 740 | [11.8] | 321 | [6.1] | 419 | [41.5] | <0.001 |
| DEMOGRAPHICS |  |  |  |  |  |  |  |
| Age [years], med (IQR) | 49.0 | [28.0; 65.0] | 49.0 | [27.0; 66.0] | 50.0 | [31.0; 62.0] | 0.557 |
| Age>65y, n (%) | 1542 | [24.6] | 1330 | [25.3] | 212 | [21.0] | 0.004 |
| Female gender, n (%) | 1601 | [25.6] | 1403 | [26.7] | 198 | [19.6] | <0.001 |
| TYPE OF INJURY |  |  |  |  |  |  |  |
| Injury type, n (%) |  |  |  |  |  |  |  |
| MVC | 858 | [13.7] | 768 | [14.6] | 90 | [8.9] |  |
| MCC | 720 | [11.5] | 638 | [12.2] | 82 | [8.1] |  |
| Bike | 458 | [7.3] | 426 | [8.1] | 32 | [3.2] |  |
| CVP | 335 | [5.4] | 301 | [5.7] | 34 | [3.4] |  |
| Sky activity | 177 | [2.8] | 94 | [1.8] | 83 | [8.2] |  |
| Hiking/Climbing | 406 | [6.5] | 175 | [3.3] | 231 | [22.9] |  |
| Winter sports | 325 | [5.2] | 209 | [4.0] | 116 | [11.5] |  |
| Work-related | 753 | [12.0] | 644 | [12.3] | 109 | [10.8] |  |
| Household | 1525 | [24.4] | 1454 | [27.7] | 71 | [7.0] |  |
| Other | 702 | [11.2] | 541 | [10.3] | 161 | [16.0] | <0.001 |
| PATIENT CHARACTERISTICS |  |  |  |  |  |  |  |
| Breathing, n (%) | 3498 | [55.9] | 3489 | [66.5] | 9 | [0.9] | <0.001 |
| Heart actions, n (%) | 4841 | [77.3] | 4825 | [91.9] | 16 | [1.6] | <0.001 |
| Circulation, n (%) | 4325 | [69.1] | 4314 | [82.2] | 11 | [1.1] | <0.001 |
| GCS, med (IQR) | 6.0 | [3.0; 14.0] | 8.0 | [3.0; 14.0] | 3.0 | [3.0; 3.0] | <0.001 |
| Docum. GCS<9, n (%) | 3602 | [57.5] | 2615 | [49.8] | 987 | [97.8] | <0.001 |
| INJURY CHARACTERISTICS |  |  |  |  |  |  |  |
| TBI, n (%) | 4281 | [68.4] | 3581 | [68.2] | 700 | [69.4] | 0.466 |
| Thorax trauma, n (%) | 1881 | [30.1] | 1503 | [28.6] | 378 | [37.5] | <0.001 |
| Abdomen trauma, n (%) | 1030 | [16.5] | 845 | [16.1] | 185 | [18.3] | 0.079 |
| Pelvis trauma, n (%) | 829 | [13.2] | 676 | [12.9] | 153 | [15.2] | 0.050 |
| Upper extremity trauma, n (%) | 960 | [15.3] | 868 | [16.5] | 92 | [9.1] | <0.001 |
| Lower extremity trauma, n (%) | 1273 | [20.3] | 1074 | [20.5] | 199 | [19.7] | 0.595 |
| DURATIONS |  |  |  |  |  |  |  |
| Response time [min], med (IQR) | 20.0 | [15.0; 26.0] | 19.0 | [15.0; 24.0] | 22.0 | [16.0; 35.0] | <0.001 |
| On scene time [min], med (IQR) | 29.0 | [22.0; 41.0] | 28.0 | [21.0; 37.0] | 48.0 | [30.0; 70.0] | <0.001 |

Abbreviations: AVP, auto versus pedestrian; GCS, Glasgow Coma Scale; IQR, interquartile range; MCC, motorcycle crash; med, median; min, minutes; MVC, motor vehicle crash; TBI, traumatic brain injury; WE, weekend

Supplement 4. Action characteristics

|  | Total (n=5534) | | Life-threatening injury  (n=5191) | | Fatal injury  (n=343) | | P-value |
| --- | --- | --- | --- | --- | --- | --- | --- |
| Breath. monitor., n (%) | 5432 | [98.2] | 5153 | [99.3] | 279 | [81.3] | <0.001 |
| Basic airway, n (%) | 4914 | [88.8] | 4615 | [88.9] | 299 | [87.2] | 0.325 |
| Adv. airway, n (%) | 2973 | [53.7] | 2746 | [52.9] | 227 | [66.2] | <0.001 |
| Anesthetics, n (%) | 2718 | [49.1] | 2694 | [51.9] | 24 | [7.0] | <0.001 |
| Hemodyn. monitor., n (%) | 5285 | [95.5] | 5003 | [96.4] | 282 | [82.2] | <0.001 |
| Temperature, n (%) | 577 | [10.4] | 548 | [10.6] | 29 | [8.5] | 0.217 |
| Vasc. access (iv/io), n (%) | 5337 | [96.4] | 5055 | [97.4] | 282 | [82.2] | <0.001 |
| Catecholamines, n (%) | 1194 | [21.6] | 955 | [18.4] | 239 | [69.7] | <0.001 |
| CPR, n (%) | 549 | [9.9] | 272 | [5.2] | 277 | [80.8] | <0.001 |
| Cardiac massage, n (%) | 503 | [9.1] | 245 | [4.7] | 258 | [75.2] | <0.001 |
| PTX decomp., n (%) | 251 | [4.5] | 192 | [3.7] | 59 | [17.2] | <0.001 |
| Defibrillation, n (%) | 73 | [1.3] | 39 | [0.8] | 34 | [9.9] | <0.001 |
| Bleeding control, n (%) | 2269 | [41.0] | 2217 | [42.7] | 52 | [15.2] | <0.001 |
| Pelvic belt, n (%) | 690 | [12.5] | 680 | [13.1] | 10 | [2.9] | <0.001 |
| Analgesics, n (%) | 3544 | [64.0] | 3524 | [67.9] | 20 | [5.8] | <0.001 |

Abbreviations: adv., advanced; breath., breathing; CPR, cardiopulmonary resuscitation; decomp., decompression; monitor., monitoring, PTX, pneumothorax, vasc., vascular.
